# Development of subtype specific RealTime PCR based growth competition assay for assessing the replication fitness of drug resistant viruses of Indian HIV-1 clade C

**DOI:** 10.1186/1471-2334-14-S3-O1

**Published:** 2014-05-27

**Authors:** Nitin K Hingankar, Ramesh S Paranjape, Srikanth P Tripathy

**Affiliations:** 1 National AIDS Research Institute, Pune, Maharashtra, India; 2 National JALMA Institute of Leprosy and other Mycobacterial Diseases, Agra, India

## Background

The knowledge of replication fitness of drug resistant viruses is essential for designing the rational treatment regimens. We report development of HIV-1 Indian Clade C specific assay for studying the replication fitness of drug resistant viruses.

## Methods

HIV-1 Subtype C molecular infectious clone pIndieC (Genebank AB023804) was used to construct two experimental vectors, pIndieC consInC RTWILD and pIndieC consInC RTSYNM, which are identical except for the presence of four synonymous nucleotides differences in Integrase. Standard curve was prepared for each vector using linear plasmid DNA template. The K103N, V106A, V106M, Y181C resistance mutations were placed in the pIndieC consInC RTWILD and replication fitness of each mutant was compared with that of wild type in a head to head growth competition assay. Virus supernatant harvested at day 0, 4, 8, 12 for RealTime quantitative PCR assessment. Relative fitness was estimated using online tool http://bis.urmc.rochester.edu/vFitness and significant differences in relative fitness between wild and mutant were determined with Mann Whitney test.

## Results

The slopes of standard curves for the vectors showed similar reaction efficiencies. Relative fitness of RTSYNM when competed with RTWILD was 1.01 demonstrating similar replication fitness and utility in competition assay. Three mutants showed decreased relative fitness compared to the wild type, with borderline significant difference in two, resulting in fitness order wild type>181C>K103N>V106M, whereas V106A mutant showed drastic decrease as almost non infectious.

## Conclusion

The growth competition assay developed is able to accurately measure differences in replication fitness as impact of single drug resistance mutation in Reverse Transcriptase.

